# A tale of two ferredoxins: sequence similarity and structural differences

**DOI:** 10.1186/1472-6807-6-8

**Published:** 2006-04-09

**Authors:** S Sri Krishna, Ruslan I Sadreyev, Nick V Grishin

**Affiliations:** 1 Howard Hughes Medical Institute, University of Texas Southwestern Medical Center, 5323, Harry Hines Blvd, Dallas, TX, 75390-9050, USA; 2 Department of Biochemistry, University of Texas Southwestern Medical Center, 5323, Harry Hines Blvd, Dallas, TX, 75390-8816, USA; 3 Joint Center for Structural Genomics, University of California, San Diego, La Jolla, CA, 92093-0314, USA

## Abstract

**Background:**

Sequence similarity between proteins is usually considered a reliable indicator of homology. Pyruvate-ferredoxin oxidoreductase and quinol-fumarate reductase contain ferredoxin domains that bind [Fe-S] clusters and are involved in electron transport. Profile-based methods for sequence comparison, such as PSI-BLAST and HMMer, suggest statistically significant similarity between these domains.

**Results:**

The sequence similarity between these ferredoxin domains resides in the area of the [Fe-S] cluster-binding sites. Although overall folds of these ferredoxins bear no obvious similarity, the regions of sequence similarity display a remarkable local structural similarity. These short regions with pronounced sequence motifs are incorporated in completely different structural environments. In pyruvate-ferredoxin oxidoreductase (bacterial ferredoxin), the hydrophobic core of the domain is completed by two β-hairpins, whereas in quinol-fumarate reductase (α-helical ferredoxin), the cluster-binding motifs are part of a larger all-α-helical globin-like fold core.

**Conclusion:**

Functionally meaningful sequence similarity may sometimes be reflected only in local structural similarity, but not in global fold similarity. If detected and used naively, such similarities may lead to incorrect fold predictions.

## Background

The millions of species of living organisms on earth possess billions of different proteins. This enormous diversity has evolved from a limited number of ancestral proteins, likely in the range of a few thousand domains [1,2]. An expansion of more than six orders of magnitude in the number of proteins has created a rich material for studying the laws of evolution. From a pragmatic perspective, evolutionary links between proteins offer shortcuts to gain knowledge about homologs from a few experimentally characterized representatives. Homology inferred from sequence is a powerful source of structure prediction, since homologs typically adopt similar three-dimensional structures [3]. It has been argued that due to the size and discreteness of the sequence space, detectable sequence similarity is a reflection of homology [4]. Therefore, a straightforward way to infer homology is by statistically supported sequence similarity. With a few notable exceptions of proteins possessing biased amino acid composition (low complexity, coiled-coil, transmembrane) that reduces the size of available sequence space, the sequence argument still stands today more than two decades after being proposed. Most commonly, sequence similarity implies homology and structural similarity [4]. Are any other scenarios possible?

Since evaluation of sequence similarity is statistical, with the frequency suggested by a statistical estimator (P-value, E-value), we expect false positives to appear [5-7]. The sequences of false positive hits display similarity comparable to that of homologs, but are not evolutionarily related and typically fold into different structures. Thus, similarity between such sequences is due to chance predicted by a corresponding probability assigned to a hit. Importantly, the sequence-based alignment with a false positive is not helpful for structure-function prediction.

With many more sequences and structures known today, we are accumulating examples where significant sequence similarity implies homology, but not necessarily global structural similarity [8-11]. One of the most notable instances is that of the KH domain, a small RNA-binding module, which was described from sequence before the structures became available [12]. Experimentally determined structures subsequently revealed global differences in the topology and fold of different KH domain homologs from eukaryotic and prokaryotic lineages [9]. The existence of such cases complicates homology-based structure prediction.

Can we find an example of sequence similarity between non-homologous proteins that is, nevertheless, useful for structure-function prediction and is, therefore, not a false positive? In other words, could two protein sequences arrive at their similarity independently (convergence) [13,14] instead of inheriting it from a common ancestor (divergence)?

## Results

### Analysis of ferredoxin sequences, structures and functions

**Figure 1:**
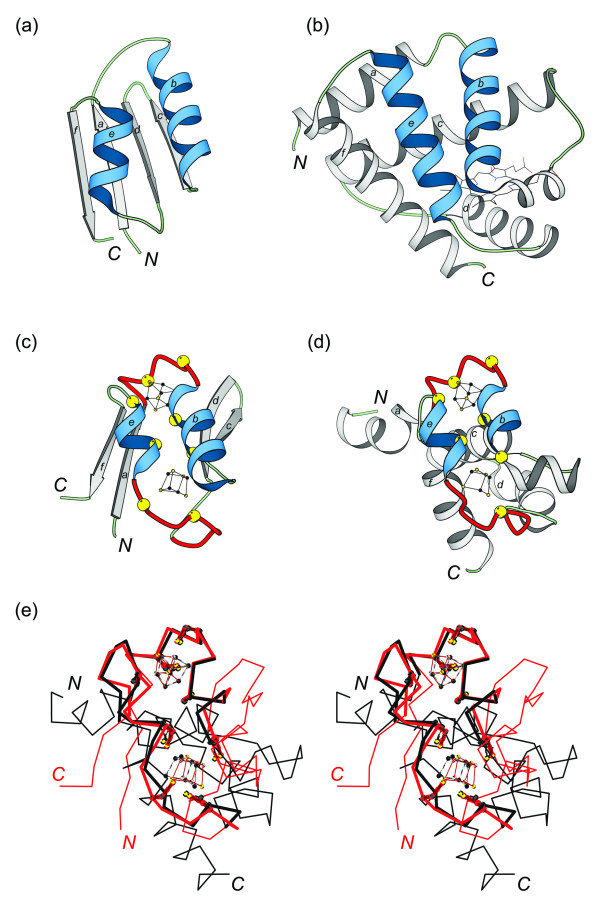
Structural comparison of the ferredoxin domains: Structural diagrams of (a) CheY-binding domain of CheA that belongs to the α+β ferredoxin-like fold (PDB: 1ffg, chain B) and (b) leghemoglobin (PDB: 2 gdm, chain A) that belongs to the globin-like fold. The structures of (c) bacterial ferredoxin domain that adopts the α+β ferredoxin-like fold and (d) α-helical ferredoxin that adopts the globin-like fold are shown. The C_α _atoms of the cysteine residues that ligate [4Fe-4S] ([3Fe-4S]) clusters are shown in CPK (yellow). The loops and helices that contribute to cluster-binding are colored red (loops) and cyan (helices), respectively. Other elements are colored grey. (e) Stereo diagram of the structural superposition of a bacterial ferredoxin (1feh, red) [36] with a α-helical ferredoxin (1kf6, black). The structures were superimposed using the program insightII by manually defining equivalent residue pairs, which are shown as thick lines (1feh, chain A: 145–157, 188–205; 1kf6, chain B: 146–158, 202–219; RMSD 0.98 Å). All figures were made using the program BOBSCRIPT [37].

Here, we suggest that the statistically supported sequence similarity found between two [Fe-S]-containing ferredoxin domains that adopt different structural folds could potentially represent such a case of analogous sequences. Ferredoxins are small electron transport proteins that are present in all organisms and adopt different structural folds. These proteins bind [Fe-S] clusters and are involved in a variety of redox reactions. The term ferredoxin refers to any electron transport protein containing one or more [Fe-S] clusters such as, for example, the [2Fe-2S] cluster-binding plant ferredoxins. In this paper, however, it is used to describe [4Fe-4S] ([3Fe-4S]) cluster-binding domains from bacterial and α-helical ferredoxin families. Bacterial ferredoxins comprise one of the largest evolutionary lineages of ferredoxins and are present both as individual proteins and as domains incorporated in larger, multi-domain proteins [15,16]. These proteins fold into a two-layer α+β structure (Fig. 1a) comprised of four β-strands and two α-helices with one or two [4Fe-4S] ([3Fe-4S]) clusters bound in their core (Fig. 1c). Helical ferredoxins similarly bind one [4Fe-4S] and one [3Fe-4S] cluster, and adopt an all-helical fold.

#### Sequence similarity searches

**Figure 2:**
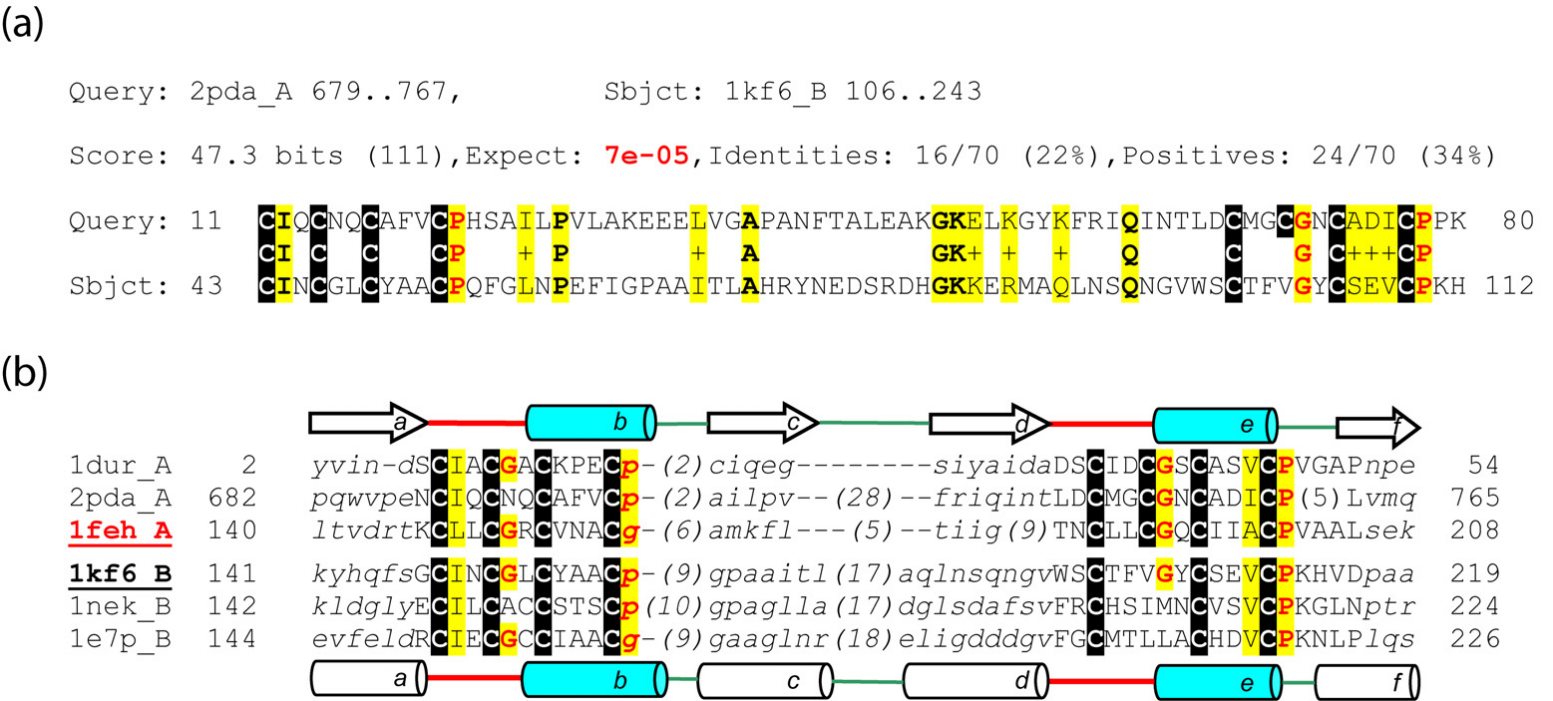
Sequence alignment of the ferredoxin domains: a) PSI-BLAST alignment of a bacterial ferredoxin domain (2pda, chain A: 679–767) with a α-helical ferredoxin domain (1kf6). The cluster-binding residues are boxed in black. Small residues near the ligand-binding site are colored red. Similar residues are colored yellow. b) Multiple structure-based sequence alignment of bacterial ferredoxins and α-helical ferredoxins. The consensus secondary structures for the two types of ferredoxins are shown: α-helices as cylinders and β-strands as arrows. The multiple alignment was made by manually defining equivalent residues for the structurally similar regions of the ferredoxin domains. Sequences shown in capitals correspond to the structurally superimposed regions. Regions that are not superimposable due to structural differences are shown in italics. The PDB code and the domain range are shown. The structures of 1feh (red) and 1kf6 (black) correspond to the superposition in figure 1e. Residues are colored according to figure 2a.

The sequence of the ferredoxin domain from *Desulfovibrio africanus *pyruvate-ferredoxin oxidoreductase (bacterial ferredoxin family, PDB: 2pda) [17] used as a PSI-BLAST [5] query detects the C-terminal ferredoxin domain of the iron protein subunit FrdB from *Escherichia coli *quinol-fumarate reductase (PDB: 1kf6, chain B) [18] in the 2^nd ^iteration with an E-value of 7E-05. The alignment spans approximately 70 residues and has two conserved motifs centered around the cysteines that bind the [Fe-S] clusters (Fig. 2a). The similarity is not limited to cysteines, but shows conservation of small residues and hydrophobic patterns as well. The use of composition-based statistics implemented in PSI-BLAST [7] is expected to compensate for the possible bias in the E-value caused by the relative abundance of the highly conserved cysteines. Indeed, when the composition-based statistics setting is not used in the last iteration (option -t F), the resulting E-value is two orders of magnitude lower (8E-07). However, one might suggest that the composition-based setting of PSI-BLAST is not a strong enough correction to decrease the cysteine influence on the search. To probe the influence of cysteines even further, we replaced the conserved cysteines with a different amino acid in both the query profile and the database sequences. Despite the absence of cysteines, PSI-BLAST produced essentially the same alignments, with somewhat higher, but still significant, E-values. For instance, replacement of Cys by Tyr (Tyr is not a very common amino acid) resulted in PSI-BLAST E-values of 0.004 and 3E-05 with and without composition-based adjustment, respectively. Replacement of Cys by Leu (Leu is a very common amino acid) resulted in E-values of 0.018 and 0.012, respectively. Thus, the sequence similarity detected by PSI-BLAST is not only caused by highly rewarded matches between cysteines, but also can be found if other amino acids are present instead.

The PSI-BLAST hits between the ferredoxins are reciprocal, and the α-helical ferredoxin sequence (1kf6) used as a query finds bacterial ferredoxins on the 2^nd ^iteration with an E-value of 0.005 (9E-05 without composition-based statistic). Similarly, an HMMer [19] search of the α-helical ferredoxin sequence (1kf6) against the Pfam database [20] using default parameters finds links to the bacterial ferredoxin family with an E-value of 7.7E-04.

#### Global structural differences

Sequence similarity statistics and functional motif conservation suggest homology and, thus, structural similarity. However, the structures of the aligned domains are globally different. Indeed, the domain from 2pda adopts a bacterial ferredoxin fold [17], which is a repeat of two βαβ units (Fig. 1a, c). This common structural motif termed the "ferredoxin-like fold" is not unique to [Fe-S]-containing bacterial ferredoxins and is observed in many other proteins that are not involved in electron transfer such as the CheY-binding domain of CheA (Fig. 1a) [21]. The ferredoxin domain of 1kf6 is entirely α-helical (Fig. 1d) and adopts a globin-like fold as defined in SCOP [22]. Indeed, leghemoglobin (Fig. 1b) [23] and the α-helical ferredoxin domain are both composed of seven α-helices with the same spatial arrangement.

#### Functional and local structural similarities

Despite global structural differences, both ferredoxin domains (2pda and 1kf6) have similar functions. The α-helical domain in quinol-fumarate reductase (1kf6) [18] is also a ferredoxin that binds one [4Fe-4S] and one [3Fe-4S] cluster similar to bacterial ferredoxin (2pda) [17] and is termed α-helical ferredoxin in SCOP [22]. Both the α-helical and bacterial ferredoxins are involved in electron transport. Helical ferredoxins participate in electron transfer pathways of the respiratory complexes succinate dehydrogenase and fumarate reductase. Bacterial ferredoxins mediate electron transfer in a variety of metabolic reactions.

While bacterial ferredoxins often bind two [4Fe-4S] clusters, some members are also known to bind [3Fe-4S] clusters. Similarly, members of the α-helical ferredoxin family bind one [4Fe-4S] and one [3Fe-4S] cluster. The cluster-binding site of these ferredoxins is formed by two cysteine-rich regions of the polypeptide chain that adopt similar conformations. The first [4Fe-4S] cluster is ligated by the cysteine side-chains of a canonical [Fe-S]-binding CXXCXXC motif that adopts a loop conformation and its structure is constrained by the integration of its cysteine sulfur atoms into the [4Fe-4S] cluster. This loop is rigidly connected via the [4Fe-4S] cluster to a α-helical segment from the second cysteine-rich region that contributes the fourth cysteine. The binding region of [3Fe-4S] possesses a similar geometry, with the cluster ligated by three cysteine side-chains, two from the canonical [Fe-S]-binding CXXCXXC motif and one from the α-helical segment. Both ferredoxins display an internal, pseudo-two-fold symmetry that relates their cluster-binding sites. For example, in the structure of the bacterial ferredoxin domain (2pda), which binds two [4Fe-4S] clusters, the cluster-chelating residues are C689, C692 and C695 from the cysteine-rich loop and C755 from the α-helical segment, for the first site, and residues C745, C748 and C751 from the complementing loop and C699 from a α-helix, for the second site. In the α-helical ferredoxin domain (1kf6), the [4Fe-4S] cluster-binding site is similarly formed by residues C148, C151 and C154 of the loop and C214 from the α-helical segment, while residues C204 and C210 from the loop and C158 from the α-helical segment contribute to the [3Fe-4S]-binding site. The presence of the [Fe-S] clusters dictates the local, and perhaps the global, structure of these ferredoxins. Removal of these clusters in bacterial ferredoxin results in an apo-ferredoxin that has no detectable secondary structure [24].

Notably, the cysteines ligating the [Fe-S] clusters in α-helical and bacterial ferredoxins are correctly aligned by PSI-BLAST. Thus, the hit between these structurally distinct domains is not a false-positive and the alignment has a predictive value. Indeed, structural superposition of the cluster-binding regions in these different ferredoxins results in an RMSD of 0.98 Å over 31 C_α _atoms from each domain (Fig. 1e). This pronounced local structural similarity covers two repeats of a loop-α-helix structure (Fig. 1e, 2b). The two loop-helix structural repeats are related by a pseudo-two-fold symmetry axis and bind two [Fe-S] clusters. Comparison of this structure-based alignment (Fig. 2b) with the PSI-BLAST sequence-based alignment (Fig. 2a) reveals that both loop-helix functional regions in these ferredoxins are aligned the same way. In other words, using the PSI-BLAST alignment and the structure of one ferredoxin, it is possible to predict correctly the local arrangement of amino acid residues in the cluster-binding region of the other ferredoxin. It is particularly significant that the PSI-BLAST alignment not only predicts correctly conformations of short local segments (loop-helix), but also infers long-range contacts between these two local segments (Fig. 1c, d, e). Short local matches of secondary structural elements (e.g. α-helix to α-helix) are frequently observed in alignments with false-positive hits, whereas correct prediction of long-range interactions is usually a property of alignments between homologs. Could we interpret these results as an inference of homology?

## Discussion

### Homology versus analogy

#### Possibility of homology

Similarity in the binding regions of the [Fe-S] clusters from α-helical and bacterial ferredoxins has been mentioned in the literature [25,26], and homology has been suggested [20,25-28]. Since both structurally distinct ferredoxins are ancient proteins [29] present in a diverse set of living organisms [30] and possibly predate the origin of most folded proteins, it is conceivable that the short loop-helix segments displaying significant sequence similarity and participating in cluster-binding are the relics of an ancient peptide world [10] that were incorporated in different larger structures. Both bacterial and α-helical ferredoxins possess an internal, pseudo-two-fold symmetry that relates their cluster-binding regions, suggesting that these domains arose as a result of an ancestral gene duplication of a single [4Fe-4S]-binding motif. Therefore, the possibility that these domains (bacterial and α-helical ferredoxins) have evolved from a common ancestral loop-helix motif, which bound a single [4Fe-4S] cluster, and diverged over time to adopt entirely different folds cannot be ruled out. In addition, the structure of dihydropyrimidine dehydrogenase from *Sus scrofa *[31] (PDB: 1h7w, chain A) contains both α-helical and bacterial ferredoxin domains on the same polypeptide chain. This could be construed as additional evidence for a homologous relationship between these domains. If these ferredoxin domains are indeed homologous, they represent an example of how far structures can diverge in evolution while retaining the function.

#### Possibility of analogy

For the following reasons, we believe it is more likely that the similarities between the two ferredoxin families are a reflection of structure-function convergence to the same functional site arrangement. First, although the PSI-BLAST E-value for sequence similarity is two orders of magnitude lower than the default statistical cutoff (Fig. 2a, b), this similarity is confined mainly to motifs of several conserved cysteine residues. Richness in cysteines and general propensity for conservation of cysteines makes both ferredoxin sequences less complex and the statistics less dependable. Although sequence analysis of the ferredoxin domains with cysteines replaced by other amino acids indicates that the match is still statistically significant, the patterns of conserved positions are driven by [Fe-S] ligating requirement and conserved positions are clustered in sequence with fixed distances between them. Sequence matches between unrelated, cysteine-rich proteins have been observed previously [32].

Second, the cysteine-rich regions are incorporated into completely different structural scaffolds (Fig. 1c, d). SCOP [22], an evolutionary-based classification of protein structures, places bacterial and α-helical ferredoxins not only in different evolutionary superfamilies and different structural folds, but also in different structural classes: alpha+beta and all-alpha proteins.

Third, and perhaps the most important consideration, electron transfer via [Fe-S] clusters is a process highly sensitive to the geometry and location of the clusters. Therefore, there is little freedom in the placement of clusters in these ferredoxins. Positioning of a cluster may be viewed similar to the positioning of the catalytic triad in serine proteases, which represent a textbook example of functional analogs [11].

Fourth, putting together the aforementioned arguments, we see resemblance to a recently reported case of structural analogy between an artificial protein evolved *in vitro *from a pool of random peptides and a group of treble-clef zinc fingers [33,34]. Both analogous proteins have several alignable cysteines functioning as zinc ligands and a local region of pronounced structural similarity. If the similarity between these two ferredoxins is indeed a reflection of functional convergence, it would arguably be the most prominent example of statistically significant and structurally meaningful sequence similarity detected between analogous proteins.

## Conclusion

Regardless of evolutionary scenarios, practical implications of the ferredoxin example for protein modeling and structure prediction are clear. It is possible to find pronounced sequence similarity that is predictive of protein function, ligand-binding site and local structure, but does not imply global fold similarity. If used naively for homology modeling, PSI-BLAST sequence alignments may be interpreted to imply fold similarity, which will be incorrect in this case. Indeed, for the majority of proteins, the presence of short motifs in an alignment implies fold similarity simply because motif conservation implies homology. However, we are seeing more and more examples of protein pairs in which local sequence motifs are incorporated in globally distinct structures [8,10]. Bacterial versus α-helical ferredoxins appears to be an extreme case of a potentially analogous sequence similarity caused by the structural constraints on the arrangement of a functional site.

## Methods

The PSI-BLAST and HMMer [5,19] programs were used to detect sequence similarity between members of the bacterial and α-helical ferredoxin families. PSI-BLAST searches were performed using as query the sequences of the ferredoxin domain from *Desulfovibrio africanus *pyruvate-ferredoxin oxidoreductase (bacterial ferredoxin family, PDB: 2pda) [17] and that of the C-terminal ferredoxin domain of the iron protein subunit FrdB from *Escherichia coli *quinol-fumarate reductase (PDB: 1kf6, chain B) [18]. PSI-BLAST version 2.2.6 with default parameters were used for these searches, in particular, inclusion cutoff of h = 0.005, with and without the application of composition-based statistics. For the alignment shown in Fig. 2a, the PSI-BLAST search was first performed on the NCBI non-redundant (nr) sequence database (2,430,773 sequences; 823,264,207 total letters), using as a query the bacterial ferredoxin sequence and the profiles after the first and second iteration were saved. These profiles were then used to search against a database of sequences of all α-helical ferredoxin structures classified by the SCOP database version 1.65. The E-value for the alignment was then scaled to the size of the nr database (database size around 0.5*10^9^). Fig. 2a shows the alignment and E-value produced by the query profile that corresponds to the second PSI-BLAST iteration on the nr database. In order to probe for a possible bias in the PSI-BLAST results due to the high conservation of cysteines in the ferredoxin sequences being compared and a corresponding low background frequency in the nr database, each of the cluster-binding cysteines in both the query profiles and database sequences were replaced by leucine (experiment 1) and by tyrosine (experiment 2). PSI-BLAST searches were then run with these artificially constructed profiles and database sequences with and without the use of the composition-based statistics. HMMer search results were obtained by using as query the α-helical ferredoxin sequence (1kf6, chain B) to search against the Pfam database [20]. The search was performed on the Pfam website [35]. Structural analysis of the ferredoxin domains were performed using the program insightII (Accelrys Software Inc.).

## Authors' contributions

RIS performed the sequence similarity searches. SSK, RIS and NVG analyzed the results, and drafted the manuscript. NVG conceived the study. All authors read and approved the final manuscript.
